# Real-world treatment intensities and pathways of macular edema following retinal vein occlusion in Korea from Common Data Model in ophthalmology

**DOI:** 10.1038/s41598-022-14386-5

**Published:** 2022-06-17

**Authors:** Yongseok Mun, ChulHyoung Park, Da Yun Lee, Tong Min Kim, Ki Won Jin, Seok Kim, Yoo-Ri Chung, Kihwang Lee, Ji Hun Song, Young-Jung Roh, Donghyun Jee, Jin-Woo Kwon, Se Joon Woo, Kyu Hyung Park, Rae Woong Park, Sooyoung Yoo, Dong-Jin Chang, Sang Jun Park

**Affiliations:** 1 Department of Ophthalmology, Hallym University College of Medicine, Kangnam Sacred Heart Hospital, Seoul, South Korea; 2 Department of Biomedical Informatics, Ajou University School of Medicine, Suwon, South Korea; 3 Department of Ophthalmology, Seoul National University College of Medicine, Seoul National University Bundang Hospital, 82, Gumi-ro 173 Beon-gil, Bundang-gu, Seongnam-si, Gyeonggi-do 13620 South Korea; 4 Department of Medical Informatics, College of Medicine, The Catholic University of Korea, Seoul, South Korea; 5 Healthcare ICT Research Center, Office of eHealth Research and Businesses, Seoul National University Bundang Hospital, Seongnam, South Korea; 6 Department of Ophthalmology, Ajou University School of Medicine, Suwon, South Korea; 7 Department of Ophthalmology and Visual Science, Yeouido St. Mary’s Hospital, College of Medicine, The Catholic University of Korea, 10, 63-ro, Yeongdeungpo-gu, Seoul, 07345 South Korea; 8 Department of Ophthalmology and Visual Science, St. Vincent’s Hospital, College of Medicine, The Catholic University of Korea, Seoul, South Korea

**Keywords:** Retinal diseases, Drug therapy

## Abstract

Despite many studies, optimal treatment sequences or intervals are still questionable in retinal vein occlusion (RVO) macular edema. The aim of this study was to examine the real-world treatment patterns of RVO macular edema. A retrospective analysis of the Observational Medical Outcomes Partnership Common Data Model, a distributed research network, of four large tertiary referral centers (n = 9,202,032) identified 3286 eligible. We visualized treatment pathways (prescription volume and treatment sequence) with sunburst and Sankey diagrams. We calculated the average number of intravitreal injections per patient in the first and second years to evaluate the treatment intensities. Bevacizumab was the most popular first-line drug (80.9%), followed by triamcinolone (15.1%) and dexamethasone (2.28%). Triamcinolone was the most popular drug (8.88%), followed by dexamethasone (6.08%) in patients who began treatment with anti-vascular endothelial growth factor (VEGF) agents. The average number of all intravitreal injections per person decreased in the second year compared with the first year. The average number of injections per person in the first year increased throughout the study. Bevacizumab was the most popular first-line drug and steroids were considered the most common as second-line drugs in patients first treated with anti-VEGF agents. Intensive treatment patterns may cause an increase in intravitreal injections.

## Introduction

Retinal vein occlusion (RVO) is a vision-threatening disorder with an incidence rate of approximately 48 per 100,000 person-years^1–3^. Macular edema is the major cause of visual deterioration in patients with RVO^4^. Advances in imaging and therapeutic modalities such as optical coherence tomography (OCT) and anti-vascular endothelial growth factor (VEGF) therapy have revolutionized the diagnosis and treatment of RVO^5^. The proangiogenic protein, VEGF, and inflammatory cytokines play key roles in the pathogenesis of macular edema^6^. There are five major agents used for treatment of RVO-related macular edema: three anti-VEGF agents including bevacizumab, ranibizumab, and aflibercept, and two steroid agents including triamcinolone and dexamethasone. Representative studies including pivotal studies demonstrated the effectiveness of these treatments^6–14^. Nevertheless, treatment patterns still contain unmet clinical needs such as optimal treatment sequence or the interval of intravitreal injections, an important factor that determines treatment burden^15,16^. In addition, a low number of studies on second or third-line drugs means that clinicians must depend on their own judgement when the first-line drug is ineffective. Randomized controlled trials (RCTs) are still the gold standard. However, RCTs cannot reflect the real-world patient population, which limits their generalizability and external validity^17^. Real-world evidence influences not only clinical practice but also healthcare policies and medical resource utilization. Therefore, an evaluation of the real-world treatment patterns on macular edema following RVO helps the development of healthcare policies and allocation of healthcare resources. In this respect, the Observational Medical Outcomes Partnership (OMOP) Common Data Model (CDM) could be useful in providing large-scale real-world data for the analysis of drug utilization patterns. The OMOP CDM, a distributed research network adopted by Observational Health Data Sciences and Informatics (OHDSI), presents healthcare data from diverse sources in a consistent and standardized way^18^. OHDSI is an international collaborative aiming at creating open-source solutions that emphasize the value of observational health data through large-scale analytics^19^. The OMOP CDM adopts standardized structures, contents, and semantics of observational data. This feature permits researchers to share their analysis codes across disparate observational healthcare databases when their healthcare data are properly extracted, transformed, and loaded in accordance with the OMOP CDM^20^. A number of large-scale collaborative studies across different data sources have been conducted, and real-world evidence has been generated using OMOP CDM^21,22^. Recently, a study has been conducted using data from the OMOP CDM to analyze the real-world incidence of endophthalmitis following anti-VEGF therapy^20^.

In this study, we conducted a characterization study to examine the real-world treatment intensities and pathways of macular edema secondary to RVO during the first two years of treatment from multiple tertiary referral centers using OMOP CDM.

## Methods

### Study design and data source

This was a retrospective, observational study. Our study used four OMOP CDM databases (version 5.3.1) derived from the electronic health records (EHR) of four large tertiary referral hospitals, Seoul National University Bundang Hospital (SNUBH), Ajou University Hospital (AUH), Yeoeuido Saint Mary’s Hospital (YSMH), and Saint Vincent’s Hospital (SVH). These EHR-based OMOP CDM databases included a total of 9,202,032 patients (SNUBH, 1,734,565; AUH, 3,109,677; YSMH, 2,279,440; SVH, 2,078,350). Each analysis was performed independently and only the aggregated results were shared and analyzed. No patient-level data were pooled across sites^23^. The characteristics of each OMOP CDM are shown in Supplementary Table S1.

### Study population

**Figure 1:**
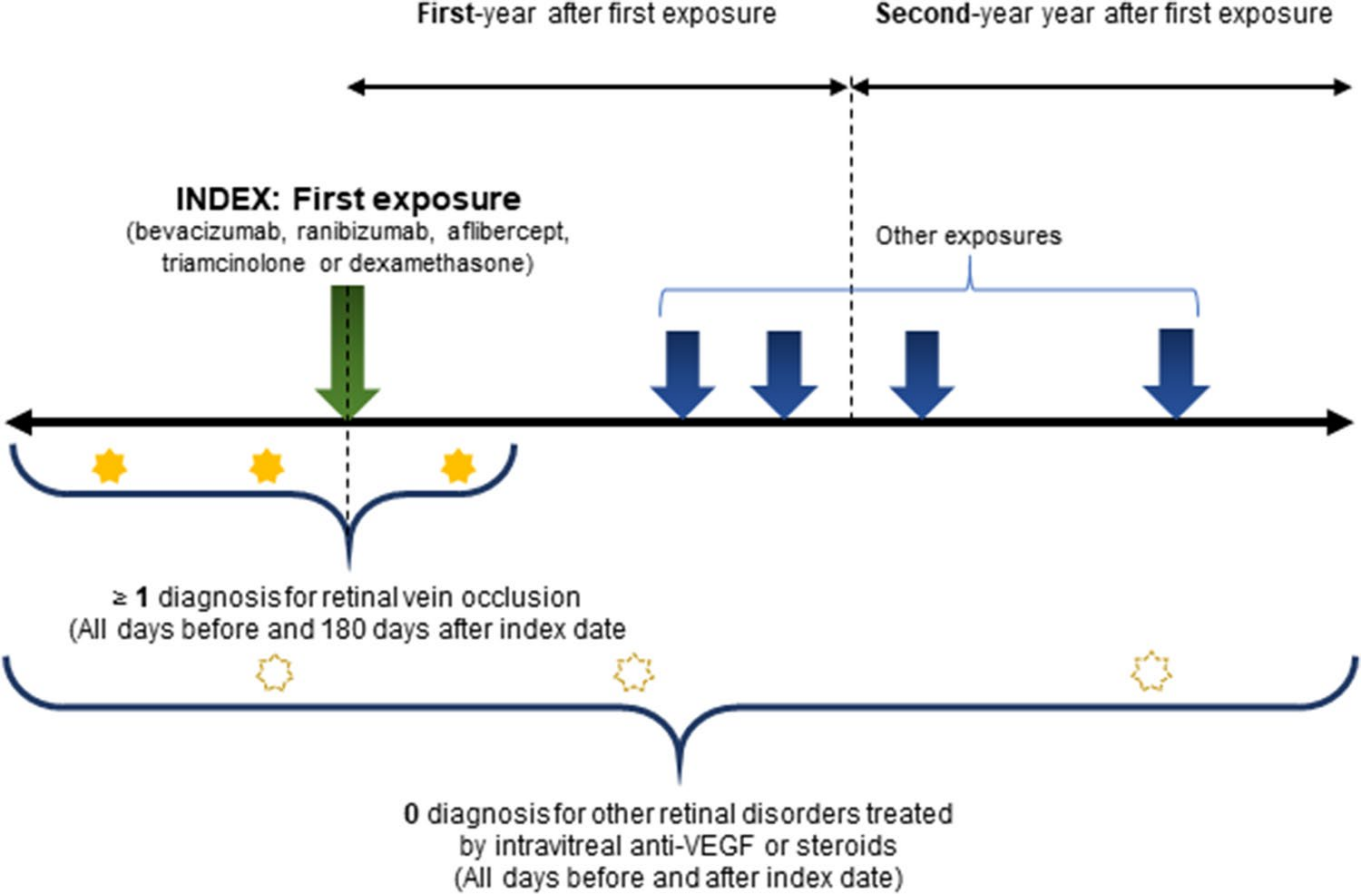
Schematic diagram for treatment pathways and intensities for macular edema following retinal vein occlusion. VEGF, vascular endothelial growth factor.

The OHDSI provides a standardized way to define and generate phenotypes through the open-source software stack, Atlas^23^. We created a retrospective, observational cohort, that included all patients diagnosed with RVO and related with five intravitreal drugs (bevacizumab, ranibizumab, aflibercept, triamcinolone, and dexamethasone). The diagnosis was coded according to the Systematized Nomenclature of Medicine Clinical Terms (SNOMED CT)^24^. We defined the index date for each case as the time of the first treatment with one of the intravitreal drugs. We only included patients with at least one diagnosis code corresponding to RVO (Supplementary Table S2) within 180 days post-index period. We excluded patients with other retinal disorders including exudative age-related macular degeneration, choroidal neovascularization due to other causes, central serous chorioretinopathy, uveitis, and inherited retinal diseases (diagnosis codes are summarized in Supplementary Table S2). Figure 1 shows a schematic diagram. The study period was from January 2003 to December 2018.

### Study outcomes

We analyzed the prescription patterns of intravitreal drugs. First, we evaluated the prescription sequences of the five target intravitreal drugs. We defined these prescription sequences as the treatment pathways^25^. To characterize these pathways, we identified the first, second, and further lines of treatment in each sequence and calculated their volume. When the drugs used as early lines of treatment were prescribed again as later lines of treatment, we did not consider them as further lines of treatment. We plotted sunburst and Sankey diagrams to display these sequences as hierarchical structures. Second, we assessed prescription frequencies in the first and second years after the initial treatment. We defined this as the treatment intensity. We calculated the average number of intravitreal injections per patient for all patients in the first year and only for patients who were followed up for at least one year in the second year. As part of a sensitivity analysis, we recalculated the treatment intensities for patients who were followed up for at least two years. In addition, we calculated the treatment intensities based on first-line drugs to determine whether the first-line drug was prescribed continuously or switched to other drugs. As the beginning of the anti-VEGF era drastically changed the treatment pattern, we divided the analysis period into three sections based on introduction of anti-VEGF drugs. The first period was before December 2005, when anti-VEGF was rarely used (January 2003–December 2005) since the off-label bevacizumab first started being used in 2006. The third period was after January 2012, since ranibizumab, first on-label anti-VEGF drugs for RVO macular edema, was approved in 2012 (January 2012–December 2018). The second period was from January 2006 to December 2011.

### Data analysis

OHDSI develops validated methodologies to aggregate results from multiple sites into a single answer to produce real-world evidence^23^. Privacy was maintained by retaining protected information of data nodes within their firewalls^25^. In this study, we used the open-source R version 3.6.3 (R Foundation for Statistical Computing, Vienna, Austria), sunburstR package version 2.1.6 (https://cran.r-project.org/web/packages/sunburstR/index.html), and networkD3 package version 0.4 (https://cran.r-project.org/web/packages/networkD3/index.html) for treatment pathway^26^. We also used the standard query language for treatment intensity. The source codes we had modified or developed were distributed and run locally, and only aggregate results were returned centrally (https://github.com/ophthal-cdm/SNUBH_RVO_TxPatternAndBurden)^19,25^.

### Ethics

This study was conducted in accordance with the tenets of the Declaration of Helsinki. Each center obtained approval from the Seoul National University Institutional Review Board (IRB) (IRB# X-1907-555-904), Ajou University Hospital IRB (IRB# RB-MED-MDB-20-271), and Catholic Medical Center (CMC) IRB (IRB# XC21RIDI0132, CMC included YSMH and SVH). The waiver of informed consent was also approved as the OMOP CDM contains only de-identified data and this study was retrospective.

## Results

### Participants

**Figure 2:**
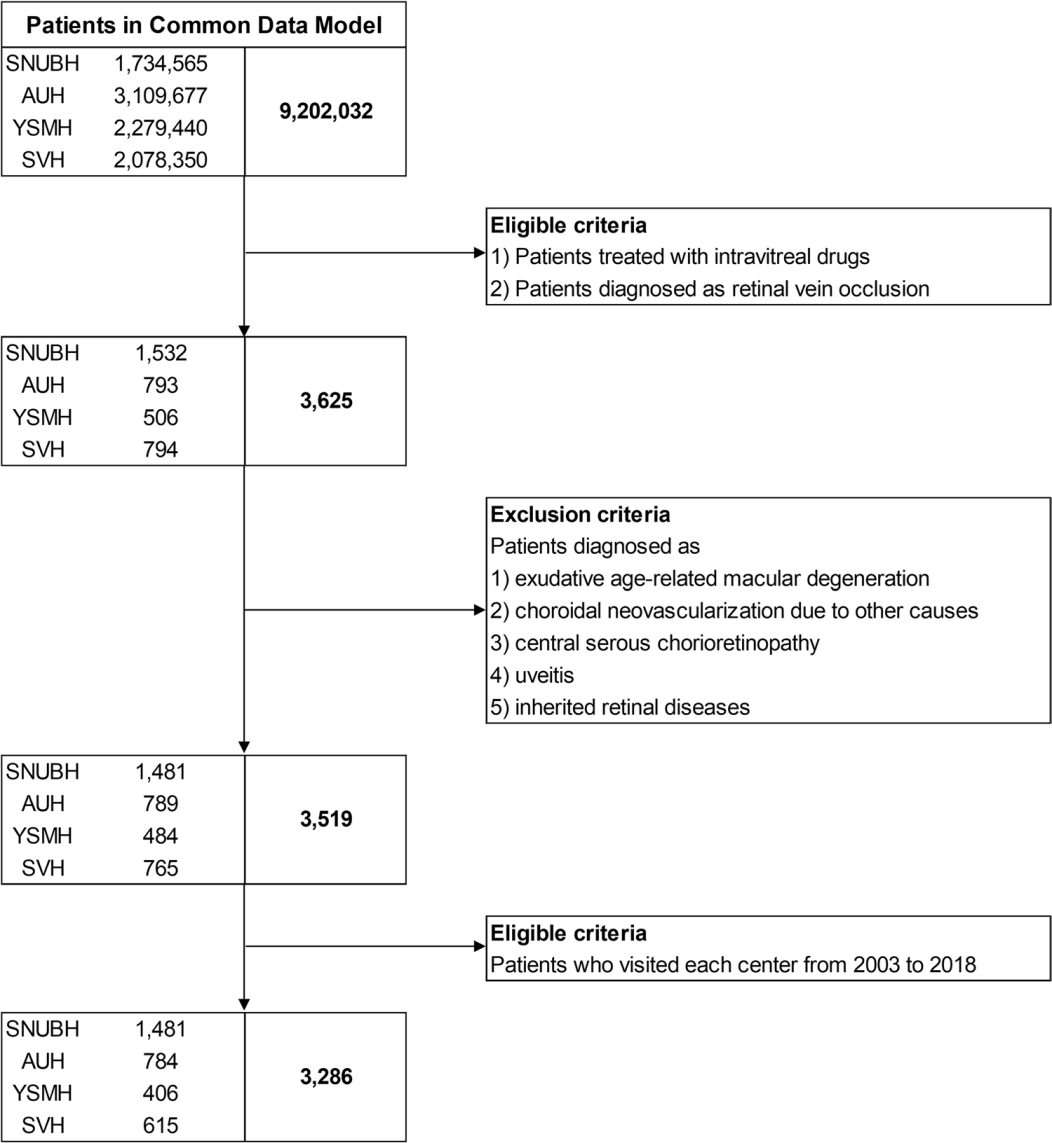
Patient selection flowchart. SNUBH, Seoul National University Bundang Hospital; AUH, Ajou University Hospital; YSMH, Yeoeuido Saint Mary’s Hospital; SVH, Saint Vincent’s Hospital.

Figure 2 shows the patient selection flowchart. We included a total of 3286 patients (mean age at first exposure; 62.9 ± 12.0 years), among which there were 1725 patients who had > 2 years of follow-up. The baseline characteristics of each group at first exposure are shown in Supplementary Table S3.

### Treatment pathway

**Figure 3:**
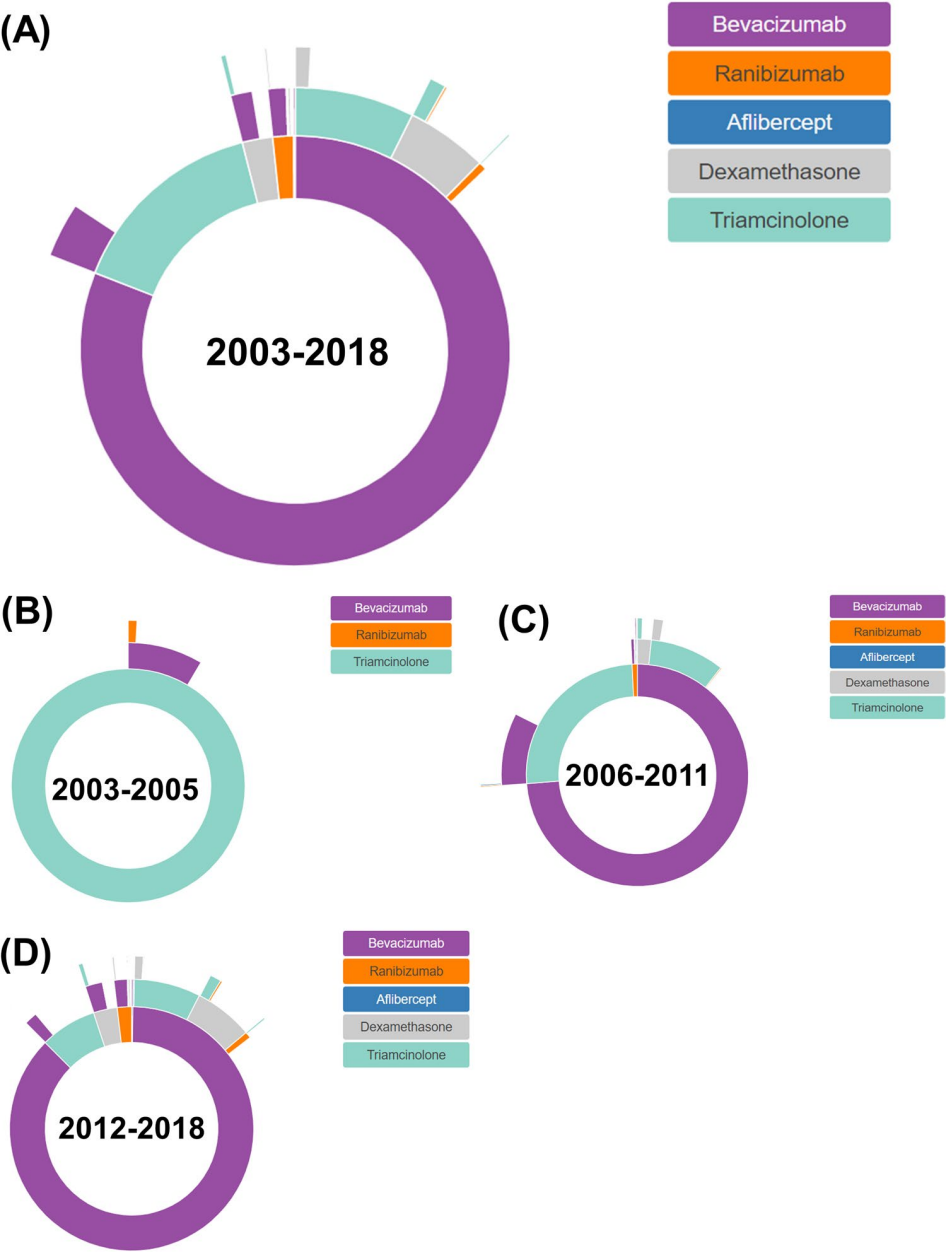
Sunburst diagrams of treatment pathways for macular edema following retinal vein occlusion. (**A**) Entire period, (**B**) January 2003–December 2005, (**C**) January 2006–December 2011, (**D**) January 2012–December 2018. R (version 3.6.3, https://cran.r-project.org) and sunburstR package (version 2.1.6, https://cran.r-project.org/web/packages/sunburstR/index.html) are used to generate this figure.

**Figure 4:**
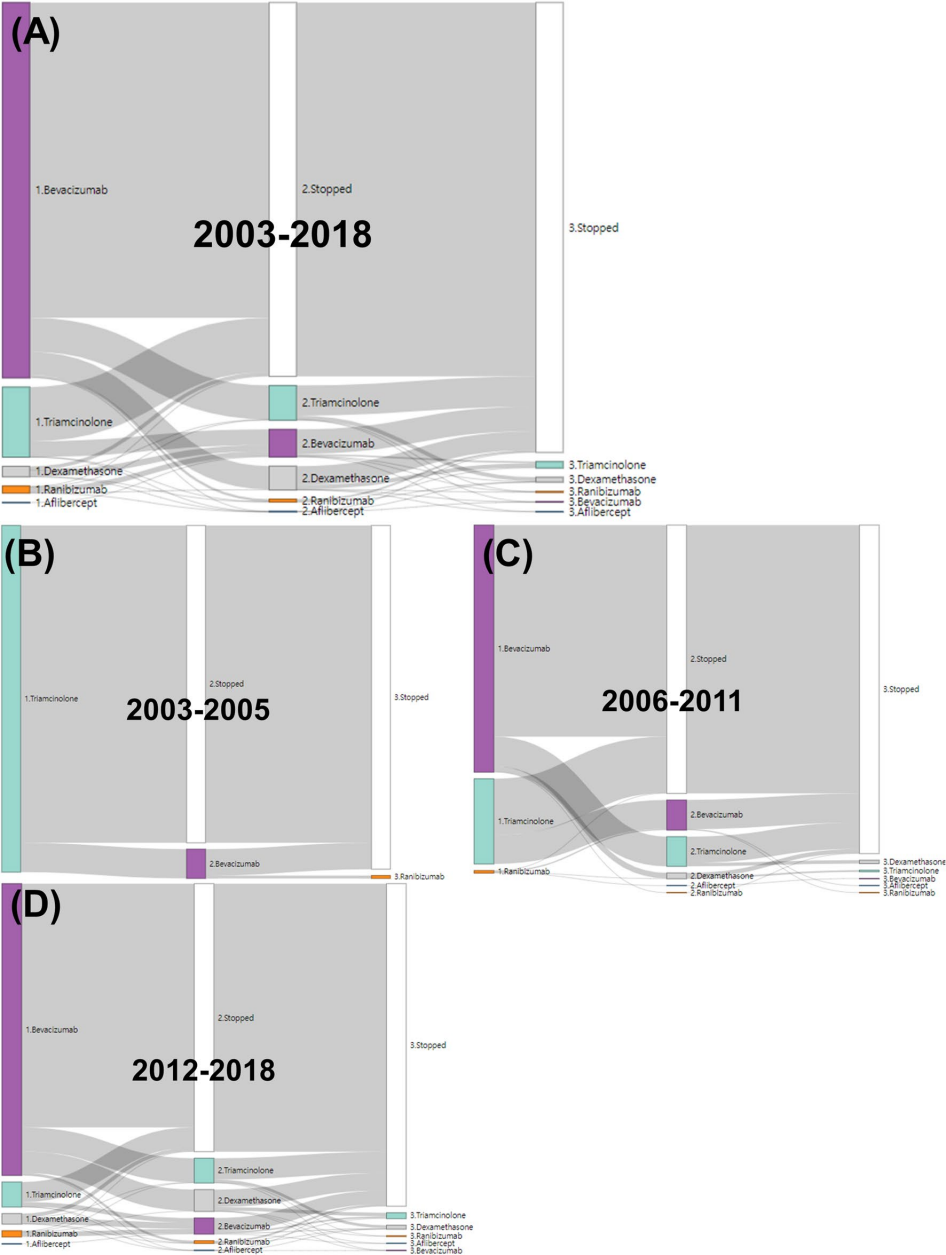
Sankey diagrams of treatment pathways for macular edema following retinal vein occlusion. (**A**) Entire period. (**B**) January 2003–December 2005, (**C**) January 2006–December 2011, (**D**) January 2012–December 2018. R (version 3.6.3, https://cran.r-project.org) and networkD3 package (version 0.4, https://cran.r-project.org/web/packages/networkD3/index.html) are used to generate this figure.

We identified 39 unique treatment pathways for RVO macular edema throughout the study period. Figures 3A and 4A present the treatment pathways from first-line to third-line treatments. Bevacizumab was the most popular first-line drug (80.9%), followed by triamcinolone (15.1%), dexamethasone (2.28%), and ranibizumab (1.55%). Of the first-line drugs, 81.0% were continued and 19.0% were discontinued in favor of second-line drugs for treating macular edema following RVO. Of the second-line drugs, 87.5% were continued and 12.5% were discontinued in favor of to third-line drugs. In patients treated with anti-VEGF drugs first, intravitreal steroids were the most common second-line drugs (total, 15.0%; triamcinolone, 8.88%; dexamethasone, 6.08%). In patients who began treatment with bevacizumab, the most prescribed second-line drug was triamcinolone (9.06%), followed by dexamethasone (6.21%). Likewise, in patients who began treatment with triamcinolone, bevacizumab (22.4%) was the most popular second-line drug. Switching to a second anti-VEGF drug occurred in 2.14% of patients who were treated with an anti-VEGF drug initially. Ranibizumab was the most common second-line anti-VEGF drug in patients who began treatment with bevacizumab, and vice versa (Fig. 3A). No patients treated with steroids as first-line drugs were switched to another steroid as a second-line treatment. Supplementary Figure S1 and 2 show the treatment pathways for each center. In all centers, the most commonly prescribed first-line drug was bevacizumab. Ranibizumab was not prescribed in Saint Vincent’s Hospital.

Figures 3B–D and 4B-D show the treatment pathways in three periods. Before December, 2005 (118 patients), triamcinolone was solely prescribed as the first-line treatment. Only bevacizumab and ranibizumab were prescribed as second-line and third-line drugs, respectively. Between January, 2006 and December, 2011 (790 patients), bevacizumab (73.8%) was the most prescribed first-line drug, followed by triamcinolone (25.4%). The most prescribed second-line drug was triamcinolone (12.0%) in patients who began treatment with bevacizumab. Likewise, the most frequently prescribed second-line drug was bevacizumab (33.9%) in patients who began treatment with triamcinolone. After January, 2012 (2378 patients), the most prescribed first-line drug was bevacizumab (87.3%), followed by triamcinolone (7.49%). Unlike other study periods, dexamethasone (7.32%) and triamcinolone (8.24%) were almost equally considered as second-line drugs in patients who began treatment with bevacizumab. The most prescribed second-line drug was bevacizumab (30.5%) in patients who began treatment with steroids.

### Treatment intensity

Table 1 shows the treatment intensity of macular edema following RVO in all patients, and Table 2 shows the results of our sensitivity analysis of patients who completed a 2-year follow-up. The treatment intensity in the second year dramatically decreased compared with that in the first year. For example, the average number of all intravitreal injections per person decreased from 1.36 to 0.29, from 2.15 to 0.50, and from 2.73 to 0.88 in the first, second, and third periods, respectively (Table 1). In contrast, the treatment intensity in the first year gradually increased over time. The average number of all intravitreal injections per person in the first year increased from 1.36 in the first period to 2.73 in the third period (Table 1). First-line drugs were maintained for subsequent treatment in most of the patients. In the third period, for example, 1,378 patients who initially began treatment with an anti-VEGF drug were still receiving anti-VEGF treatment in the second year, while only 186 patients had switched to steroids (Table 1). The same trend was observed for the steroids. The use of dexamethasone implants increased during the third period.

**Table 1:** Average number of intravitreal injections in all treated patients with macular edema following retinal vein occlusion.

	Anti-VEGF as first drug		Steroid as first drug		All drugs	
	First year	Second year	First year	Second year	First year	Second year
**January, 2003 ~ December, 2005**						
All drugs	NA	NA	1.36 (n = 118)	0.29 (n = 94)	1.36 (n = 118)	0.29 (n = 94)
Anti-VEGF						
Bevacizumab	NA	NA	1.00 (n = 1)	2.20 (n = 1)	1.00 (n = 1)	2.20 (n = 1)
Ranibizumab	NA	NA	NA	NA	NA	NA
Aflibercept	NA	NA	NA	NA	NA	NA
Steroid						
Dexamethasone	NA	NA	NA	NA	NA	NA
Triamcinolone	NA	NA	1.35 (n = 118)	0.17 (n = 94)	1.35 (n = 118)	0.17 (n = 94)
**January, 2006 ~ December, 2011**						
All drugs	2.31 (n = 589)	0.53 (n = 477)	1.68 (n = 201)	0.38 (n = 166)	2.15 (n = 790)	0.50 (n = 643)
Anti-VEGF						
Bevacizumab	2.22 (n = 585)	0.47 (n = 474)	1.79 (n = 43)	1.10 (n = 42)	2.20 (n = 628)	0.52 (n = 516)
Ranibizumab	1.67 (n = 6)	0.25 (n = 4)	3.00 (n = 1)	4.00 (n = 1)	1.86 (n = 7)	1.00 (n = 5)
Aflibercept	NA	NA	NA	NA	NA	NA
Steroid						
Dexamethasone	1.00 (n = 2)	1.50 (n = 2)	NA	NA	1.00 (n = 2)	1.50 (n = 2)
Triamcinolone	1.24 (n = 38)	0.61 (n = 33)	1.28 (n = 201)	0.08 (n = 166)	1.28 (n = 239)	0.16 (n = 199)
**January, 2012 ~ December, 2018**						
All drugs	2.82 (n = 2122)	0.94 (n = 1341)	1.94 (n = 256)	0.49 (n = 193)	2.73 (n = 2378)	0.88 (n = 1534)
Anti-VEGF						
Bevacizumab	2.65 (n = 2109)	0.78 (n = 1331)	1.97 (n = 68)	1.18 (n = 55)	2.63 (n = 2177)	0.80 (n = 1386)
Ranibizumab	2.02 (n = 58)	0.63 (n = 43)	2.67 (n = 3)	0 (n = 3)	2.05 (n = 61)	0.59 (n = 46)
Aflibercept	1.62 (n = 8)	0.25 (n = 4)	1.00 (n = 2)	NA	1.50 (n = 10)	0.25 (n = 4)
Steroid						
Dexamethasone	1.20 (n = 122)	0.87 (n = 104)	1.45 (n = 77)	0.20 (n = 60)	1.30 (n = 199)	0.63 (n = 164)
Triamcinolone	1.30 (n = 102)	1.13 (n = 82)	1.29 (n = 186)	0.13 (n = 137)	1.29 (n = 288)	0.51 (n = 219)

VEGF, vascular endothelial growth factor.

**Table 2:** Average number of intravitreal injections in patients observed for at least 2 years and treated with macular edema following retinal vein occlusion.

	Anti-VEGF as first drug		Steroid as first drug		All drugs	
	First year	Second year	First year	Second year	First year	Second year
**January, 2003 ~ December, 2005**						
All drugs	NA	NA	1.45 (n = 86)	0.30 (n = 86)	1.45 (n = 86)	0.30 (n = 86)
Anti-VEGF						
Bevacizumab	NA	NA	1.00 (n = 1)	2.20 (n = 1)	1.00 (n = 1)	2.20 (n = 1)
Ranibizumab	NA	NA	NA	NA	NA	NA
Aflibercept	NA	NA	NA	NA	NA	NA
Steroid						
Dexamethasone	NA	NA	NA	NA	NA	NA
Triamcinolone	NA	NA	1.44 (n = 86)	0.18 (n = 86)	1.44 (n = 86)	0.18 (n = 86)
**January, 2006 ~ December, 2011**						
All drugs	2.45 (n = 420)	0.58 (n = 420)	1.84 (n = 149)	0.39 (n = 149)	2.29 (n = 569)	0.53 (n = 569)
Anti-VEGF						
Bevacizumab	2.35 (n = 418)	0.51 (n = 418)	1.77 (n = 39)	1.08 (n = 39)	2.30 (n = 457)	0.56 (n = 457)
Ranibizumab	2.00 (n = 3)	NA (n = 3)	3.00 (n = 1)	4.00 (n = 1)	2.25 (n = 4)	1.00 (n = 4)
Aflibercept	NA	NA	NA	NA	NA	NA
Steroid						
Dexamethasone	1.00 (n = 2)	1.00 (n = 2)	NA	NA	1.00 (n = 2)	1.00 (n = 2)
Triamcinolone	1.29 (n = 31)	0.65 (n = 31)	1.35 (n = 149)	0.09 (n = 149)	1.34 (n = 180)	0.18 (n = 180)
**January, 2012 ~ December, 2018**						
All drugs	3.12 (n = 928)	1.01 (n = 928)	1.86 (n = 142)	0.54 (n = 142)	2.95 (n = 1070)	0.95 (n = 1070)
Anti-VEGF						
Bevacizumab	2.92 (n = 925)	0.84 (n = 925)	1.64 (n = 41)	1.39 (n = 41)	2.86 (n = 966)	0.86 (n = 966)
Ranibizumab	1.88 (n = 16)	0.81 (n = 16)	2.00 (n = 1)	0 (n = 1)	1.88 (n = 17)	0.77 (n = 17)
Aflibercept	4.00 (n = 1)	NA (n = 1)	NA	NA	4.00 (n = 1)	NA (n = 1)
Steroid						
Dexamethasone	1.17 (n = 70)	0.99 (n = 70)	1.48 (n = 44)	0.20 (n = 44)	1.29 (n = 114)	0.68 (n = 114)
Triamcinolone	1.31 (n = 62)	1.20 (n = 62)	1.30 (n = 100)	0.11 (n = 100)	1.30 (n = 162)	0.53 (n = 162)

VEGF, vascular endothelial growth factor.

## Discussion

Using the OMOP CDM data, we were able to identify the treatment pathways and intensities of patients diagnosed with macular edema following RVO. We found that, with the introduction of intravitreal anti-VEGF, retinal specialists had a high preference for anti-VEGF drugs as a first-line treatment, with bevacizumab being the most popular choice. In the first and second periods, triamcinolone was prescribed most frequently among the steroids. However, the volume of dexamethasone implant prescriptions had increased steadily after its approval for macular edema secondary to RVO (Table 1)^4^. Intravitreal steroids were generally considered as a second-line drug for macular edema patients treated with anti-VEGF drugs first. Intravitreal triamcinolone and dexamethasone implants had almost equal proportions as second-line drugs for patients who received bevacizumab as a first-line drug (Fig. 3). The average number of intravitreal injections in the second year was lower than that in the first year. Additionally, the number of intravitreal injections increased throughout the course of the first year (Table 1).

Several studies have found that anti-VEGF drugs and dexamethasone implants have comparable efficacies for treating macular edema in RVO patients^27,28^. However, the European Society of Retina Specialists also recommend anti-VEGF agents as a first-line drug, and the data we examined in this study shows this recommendation to be a popular choice^5^. The side effects of steroids have most likely caused physicians consider anti-VEGF agents as a first-line drug. Moderate-certainty evidence from a Cochrane systematic review has suggested that there is an increased risk of cataracts and raised intraocular pressure associated with steroids compared with anti-VEGF drugs^29^. Despite its off-label clinical use, bevacizumab was the most popular agent for treating macular edema following RVO, potentially because of its relatively low cost^30^. Several studies have reported the noninferiority of bevacizumab to ranibizumab or aflibercept^7,31–33^.

Patients initially treated with anti-VEGF drugs were given intravitreal injections of triamcinolone and dexamethasone implants in 7.32% and 8.24% of cases, respectively. Considering that the dexamethasone implant was introduced later than triamcinolone as a treatment for macular edema related to RVO, it is worth noting the greater popularity of dexamethasone implants as a second-line drug that triamcinolone^13,14,34^.

We found that ranibizumab was prescribed as a second-line anti-VEGF therapy in patients treated with bevacizumab as a first-line drug, and vice versa. A patient being switched from ranibizumab to bevacizumab might be related to national health insurance policy, because full coverage could not be provided in cases of inadequate response to ranibizumab. In these cases, physicians may have had to consider bevacizumab to reduce the financial burden on patients. Bevacizumab sometimes had a very high priority for a similar reason. Cases where patients were switched from bevacizumab to ranibizumab might originate from the approval of ranibizumab and a poor response to bevacizumab.

A decrease in the average number of injections in the second year suggests that macular edema could, in many cases, be successfully resolved with only the first injection. Poor response to the treatment, missed visits, or use of other treatment modalities rather than intravitreal injection could also contribute the reduced number of injections. However, the results of previous studies support the former conclusion. A large retrospective study of pro-re-nata treatment reported that patients with RVO received a median of six anti-VEGF injections in the first year and fewer injections in subsequent years^35^. Follow-up extension research on a pivotal study for aflibercept in central retinal vein occlusion (CRVO) reported that the mean number of injections decreased from 2.5 (between weeks 24 and 52) to 1.3 (between weeks 52 and 76)^6^. Long term results of pivotal studies for ranibizumab in branch retinal vein occlusion (BRVO) and CRVO found that the mean number of injections decreased from 3.1 (between months 6 and 11) to 1.3 (second year) and from 4.0 (between months 6 and 11) to 2.25 (second year), respectively^36^. Other real-world studies about RVO macular edema represented similar tendencies. A study of 5,661 patients with BRVO macular edema reported that treatment visits per patient were 5.1 and 1.3 in the first and second year, respectively (total treatment visits per patient: 5.1 at 12 months and 6.4 at 24 months)^37^. Another study of 8,876 patients with BRVO macular edema showed that the mean number of anti-VEGF injections decreased from 4.5 to 2.9 from the first 6 months to the second 6 months^38^. This study also revealed that the mean number of anti-VEGF injections decreased from 4.6 to 3.0 in 6737 patients with CRVO macular edema^38^.

The mean number of injections in the first year increased over time, especially for anti-VEGF drugs, whose popularity also increased over the course of the year. Pivotal studies for ranibizumab and aflibercept, and studies on the efficacy of bevacizumab have been linked to the increased use of anti-VEGF drugs^6–10^. Furthermore, the effectiveness of monthly consecutive injections for at least 3 months has been widely accepted among physicians in neovascular age-related macular degeneration^39,40^. A multicenter, retrospective study revealed that this dosing regimen was also maintained in macular edema following RVO^41^. Therefore, intensive practice patterns might cause an increase in intravitreal injections.

It should be noted that the popularity of the off-label drug bevacizumab in our study, far exceeded that of ranibizumab, aflibercept, or dexamethasone (which are all on-label drugs for macular edema in RVO). The efficacy and safety of on-label drugs has been already been proven by level 1 evidence research: prospective, randomized, and controlled studies. Nevertheless, for the patients in this study, the most popular first-line drug for macular edema secondary to RVO was bevacizumab. Another real-world report on RVO macular edema revealed that a majority of patients received ranibizumab (51.3%), with bevacizumab (44.1%) and aflibercept (4.6%) as the next most popular choices^41^. In our country, all medical services are provided under the National Healthcare Insurance Act and “covered services” allow patients to pay only part of the cost. Although covered services include treatments with on-label drugs for macular edema following RVO, their use is regulated by several conditions, such as minimum macular thickness, visual acuity, the limitation of the number of injections, or response to treatment. This makes physicians hesitant to use on-label drugs and provide an incentive to choose bevacizumab, which is free from these constraints. The noninferiority of bevacizumab might also ensures that it remains a valid choice of treatment^31^. In addition, the lower cost of bevacizumab compared with other agents is likely one of the most important factors for physicians and patients to prefer it. A sharp increase in the use of dexamethasone implants was in line with the influence of national insurance policies, because of the deregulation in the number of dexamethasone implant injections in 2019.

Real-world practice patterns are influenced not only by the introduction of drugs and their efficacies, but also by the real-world environment, such as government policies, cost, resources, or burden related to the treatment. A public health policy maker should consider these aspects. If gaps between pivotal studies and real-world practice patterns persist, iterative studies are necessary to determine the best course of action in a given real-world environment. Recently, many real-world studies, including registry studies, have been conducted to provide real-world evidence. The results of these studies are reflected in clinical practice guidelines^5,11,42^. Our study, which examined real-world patient data, provides valuable real-world evidence and practice patterns for the treatment of RVO-related macular edema.

This study is a multicenter, large-scale, and real-world study that can be expanded further at any time owing to the scalability of CDM. Furthermore, it identifies real-world practice patterns and visualizes the treatment pathways and intensities of macular edema secondary to RVO. However, there are several limitations to this study because of its retrospective nature. First, we did not distinguish between CRVO and BRVO because information loss occurred during mapping of the original diagnostic data to SNOMED CT. Moreover, it is also difficult to distinguish whether the macular edema is associated with retinal vein occlusion or diabetes (i.e. diabetic macular edema) when retinal vein occlusion and macular edema exist together in patients with diabetes. We considered them as patients with macular edema following retinal vein occlusion inevitably. Second, the parameters of visual outcomes (such as visual acuity or findings from optical coherence tomography) could not be collected because the results of ophthalmic examination are still being extracted, transformed and loaded into the CDM^43^. Many subsequent studies using these data will be conducted and reported soon. Lack of information on special situations, such as a patient switching to another drug for nonmedical reasons, is another limitation because the CDM restricts the review of individual records. In addition, a history of focal, grid laser, or vitrectomy cannot be collected because not all hospitals have uploaded records related to previous procedures into the CDM. Further studies including these procedures will be conducted in the near future.

In conclusion, bevacizumab was the most popular prescription as a first-line drug, and its volume has increased over time. Triamcinolone and dexamethasone were most popularly chosen as second-line drugs, and the preference for dexamethasone implants rapidly increased over time. The CDM can reflect real-world practice patterns better than claim databases because it includes drugs not covered by health insurance. Furthermore, it is a valuable tool owing to its scalability for large-scale multicenter studies without patient-level pooled analysis; it only allows an analysis with aggregated results.

## Supplementary Information

Supplementary Information.

## Abbreviations

RVO: Retinal vein occlusion
VEGF: Vascular endothelial growth factor
OMOP: Observational medical outcomes partnership
CDM: Common data model
