# Supplementary material for: Real-world treatment intensities and pathways of macular edema following retinal vein occlusion in Korea from Common Data Model in ophthalmology
Source: Sci Rep. 2022 Jun 17;12:10162. doi: 10.1038/s41598-022-14386-5 (PMC9205933; doi:10.1038/s41598-022-14386-5)
Supplement: Supplementary file 1 — Supplementary Information. [file 41598_2022_14386_MOESM1_ESM.docx]

**Supplementary Table S1.** Overall characteristics across common data model

| **Data Source** | **No. of Patients** | **Male** | **Female** | **Start Date** | **End Date** |
| --- | --- | --- | --- | --- | --- |
| SNUBH | 1,734,565 | 828,617 | 905,948 | 2003-04-02 | 2018-12-31 |
| AUH | 3,109,677 | 1,649,109 | 1,460,568 | 1999-04-06 | 2018-12-31 |
| YSMH | 2,279,440 | 1,117,404 | 1,162,034 | 2009-01-01 | 2020-09-04 |
| SVH | 2,078,350 | 949,933 | 1,126,429 | 2009-01-01 | 2020-08-05 |

Abbreviations: SNUBH, Seoul National University Bundang Hospital; YSMH, Yeoeuido Saint Mary’s Hospital; SVH, Saint Vincent’s Hospital; AUH, Ajou University Hospital

**Supplementary Table S2**. Identifications for diagnoses and drugs

|  | **Concept Identification** | **Description** |
| --- | --- | --- |
| **Intravitreal drugs** | 1397141 | Bevacizumab |
|  | 19080982 | Ranibizumab |
|  | 40244266 | Aflibercept |
|  | 42941639, 41294127 | Triamcinolone |
|  | 40160929 | Dexamethasone |
| **Diagnosis for retial vein occlusion** | 312622 | Venous retinal branch occlusion |
|  | 313761 | Central retinal vein occlusion |
|  | 436692 | Retinal venous engorgement |
|  | 4082279 | Retinal veins beaded |
|  | 4082300 | Retinociliary vein |
|  | 4083481 | Distension of vein beyond arteriovenous crossing |
|  | 4083482 | Macular branch retinal vein occlusion |
|  | 4090239 | Retinal vein finding |
|  | 4090240 | Retinal veins dilated |
|  | 4090241 | Retinal veins sheathed |
|  | 4090242 | Retinal venous loops |
|  | 4102317 | Incipient occlusion of retinal vein |
|  | 4161670 | Venous beading of retina due to diabetes mellitus |
|  | 4187790 | Thrombosis of retinal vein |
|  | 4199035 | Branch retinal vein occlusion with no neovascularization |
|  | 4204368 | Eales' disease |
|  | 4208221 | Central retinal vein occlusion - ischemic |
|  | 4208222 | Central retinal vein occlusion - non-ischemic |
|  | 4216561 | Thrombophlebitis of retinal vein |
|  | 4224094 | Retinal venous tortuosity |
|  | 4271392 | Retinal vein appearance - finding |
|  | 4280502 | Partial occlusion of retinal vein |
|  | 4324290 | Retinal phlebitis |
|  | 4334246 | Central retinal vein occlusion with neovascularization |
|  | 4334247 | Hemispheric retinal vein occlusion |
|  | 4334248 | Branch retinal vein occlusion with neovascularization |
|  | 4334888 | Central retinal vein occlusion - juvenile with neovascularization |
|  | 4335591 | Central retinal vein occlusion - juvenile with macular edema |
|  | 4335592 | Hemispheric retinal vein occlusion with macular edema |
|  | 4336005 | Hemispheric retinal vein occlusion with neovascularization |
|  | 4339010 | Central retinal vein occlusion with macular edema |
|  | 4339011 | Central retinal vein occlusion - juvenile |
|  | 4339013 | Branch retinal vein occlusion with macular edema |
|  | 36674765 | Venous beading of right retina due to diabetes mellitus |
|  | 36674766 | Venous beading of left retina due to diabetes mellitus |
|  | 37206377 | Occlusion of branch of retinal vein of left eye |
|  | 37206378 | Occlusion of branch of retinal vein of right eye |
|  | 37206380 | Occlusion of central retinal vein of right eye |
|  | 37206381 | Occlusion of central retinal vein of left eye |
|  | 37208204 | Partial occlusion of left retinal vein |
|  | 37208205 | Partial occlusion of right retinal vein |
|  | 42535735 | Combined occlusion by thrombus of retinal artery and retinal vein |
| **Diagnosis for exclusion** | 46273984 | Cytomegalovirus chorioretinitis |
|  | 46273126 | Syphilitic iritis |
|  | 46272745 | Autosomal dominant vitreoretinochoroidopathy |
|  | 46269950 | Iridocyclitis due to tuberculosis |
|  | 45772123 | Sarcoid iridocyclitis |
|  | 45771029 | Amelogenesis imperfecta co-occurrent with cone rod dystrophy |
|  | 45770924 | Herpes zoster iritis |
|  | 45770915 | Lens induced iritis |
|  | 45757732 | Noninfectious secondary iritis |
|  | 45757712 | Gonococcal iritis |
|  | 45757706 | Macular focal choroiditis |
|  | 45757696 | Syphilitic choroiditis |
|  | 45757694 | Toxoplasma choroiditis |
|  | 45757573 | Infectious secondary iritis |
|  | 44784343 | Peripheral disseminated choroiditis |
|  | 44783868 | Granulomatous inflammatory arthritis, dermatitis and uveitis, familial |
|  | 44783673 | Focal choroiditis of posterior pole of eye |
|  | 44783665 | Peripheral focal choroiditis |
|  | 44782876 | Posterior pole disseminated choroiditis |
|  | 44782875 | Generalized disseminated choroiditis |
|  | 44782851 | Hereditary retinal dystrophy primarily involving sensory retina |
|  | 44782850 | Hereditary retinal dystrophy primarily involving retinal pigment epithelium |
|  | 42599783 | Reflex uveitis |
|  | 42599717 | Traumatic anterior uveitis |
|  | 42573169 | Equine recurrent uveitis |
|  | 40485513 | Chronic uveitis |
|  | 40480902 | Reactivation of toxoplasmosis chorioretinitis |
|  | 40480545 | Pneumocystis jirovecii choroiditis |
|  | 37396748 | Severe early childhood onset retinal dystrophy |
|  | 37395995 | Bothnia retinal dystrophy |
|  | 37395980 | MORM syndrome |
|  | 37395938 | Retinitis punctata albescens |
|  | 37312551 | Infectious panuveitis |
|  | 37312266 | Iritis co-occurrent with inflammatory bowel disease |
|  | 37312155 | Complete achromatopsia |
|  | 37312154 | Blue cone monochromatism |
|  | 37311935 | Infectious intermediate uveitis |
|  | 37311893 | Posterior uveitis due to infectious disease |
|  | 37209448 | Iritis of left eye caused by herpes zoster virus |
|  | 37209447 | Iritis of right eye caused by herpes zoster virus |
|  | 37209446 | Bilateral pars planitis |
|  | 37208203 | Bilateral neovascularization of choroid of eye |
|  | 37208019 | Acute endophthalmitis of left eye |
|  | 37208014 | Acute endophthalmitis of right eye |
|  | 37208000 | Bilateral chorioretinitis of eyes |
|  | 37207997 | Bilateral chronic iritis |
|  | 37207954 | Chorioretinitis of left eye |
|  | 37207951 | Chronic iritis of left eye |
|  | 37207894 | Chorioretinitis of right eye |
|  | 37207891 | Chronic iritis of right eye |
|  | 37204325 | Facial dysmorphism, anorexia, cachexia, eye and skin anomalies syndrome |
|  | 37203959 | Ankylosing spondylitis co-occurrent with anterior uveitis |
|  | 37118649 | Hypogonadotropic hypogonadism retinitis pigmentosa syndrome |
|  | 37117174 | Autosomal recessive bestrophinopathy |
|  | 37116578 | Noninfectious panuveitis |
|  | 37116567 | Primary anterior uveitis |
|  | 37116419 | Myopic choroidal neovascularization |
|  | 37115759 | Cleft lip retinopathy syndrome |
|  | 37115758 | X-linked intellectual disability, limb spasticity, retinal dystrophy, diabetes insipidus syndrome |
|  | 37111354 | Sporadic Blau syndrome |
|  | 37111019 | Butterfly-shaped pigmentary macular dystrophy |
|  | 37110832 | 5-amino-4-imidazole carboxamide ribosiduria |
|  | 37110788 | Aland Islands eye disease |
|  | 37110506 | Ataxia co-occurrent and due to phytanic acid storage disease |
|  | 37110039 | Autosomal recessive posterior column ataxia and retinitis pigmentosa |
|  | 37109996 | Retinitis pigmentosa, intellectual disability, deafness, hypogenitalism syndrome |
|  | 37109995 | Retinohepatoendocrinologic syndrome |
|  | 37109994 | RHYNS syndrome |
|  | 37109775 | Spastic tetraplegia, retinitis pigmentosa, intellectual disability syndrome |
|  | 37109698 | Reticular dystrophy of retinal pigment epithelium |
|  | 37109673 | Polyneuropathy, hearing loss, ataxia, retinitis pigmentosa, cataract syndrome |
|  | 37109492 | Histoplasmosis syndrome of right eye |
|  | 37109491 | Histoplasmosis syndrome of left eye |
|  | 36717573 | Optic disc swelling co-occurrent with uveitis |
|  | 36717531 | Osteochondrodysplatic nanism, deafness, retinitis pigmentosa syndrome |
|  | 36717362 | Acute iritis of right eye |
|  | 36717306 | Acute multifocal placoid pigment epitheliopathy of left eye |
|  | 36716161 | Oligocone trichromacy |
|  | 36716158 | Oculotrichodysplasia |
|  | 36715352 | Amaurosis hypertrichosis syndrome |
|  | 36715306 | Ectodermal dysplasia with ectrodactyly and macular dystrophy syndrome |
|  | 36714337 | Benign concentric annular macular dystrophy |
|  | 36714290 | Cone dystrophy with supernormal rod response |
|  | 36714278 | Autosomal dominant late-onset retinal degeneration |
|  | 36714163 | Primary ciliary dyskinesia and retinitis pigmentosa syndrome |
|  | 36714153 | Progressive bifocal chorioretinal atrophy |
|  | 36714106 | Spondylometaphyseal dysplasia with cone-rod dystrophy syndrome |
|  | 36713326 | Neovascularization of choroid of right eye |
|  | 36713324 | Neovascularization of choroid of left eye |
|  | 36713313 | Histoplasmosis syndrome of bilateral eyes |
|  | 36713148 | Acute anterior uveitis of bilateral eyes |
|  | 36713147 | Bilateral acute iritis |
|  | 36713145 | Bilateral acute multifocal placoid pigment epitheliopathy |
|  | 36713131 | Acute anterior uveitis of left eye |
|  | 36713130 | Acute iritis of left eye |
|  | 36713119 | Acute anterior uveitis of right eye |
|  | 36713117 | Acute multifocal placoid pigment epitheliopathy of right eye |
|  | 36712991 | Central serous choroidopathy of left eye |
|  | 36712990 | Central serous choroidopathy of bilateral eyes |
|  | 36712989 | Central serous choroidopathy of right eye |
|  | 36687176 | Uveitis of left eye caused by Toxoplasma gondii |
|  | 36687175 | Uveitis of right eye caused by Toxoplasma gondii |
|  | 36687174 | Uveitis of bilateral eyes caused by Toxoplasma gondii |
|  | 36686922 | Bilateral uveitis of eyes |
|  | 36686921 | Uveitis of left eye |
|  | 36686920 | Uveitis of right eye |
|  | 36684792 | Bilateral chronic anterior uveitis of eyes |
|  | 36684767 | Bilateral panuveitis of eyes |
|  | 36684732 | Chronic iridocyclitis of left eye |
|  | 36684701 | Panuveitis of left eye |
|  | 36684666 | Chronic anterior uveitis of right eye |
|  | 36684637 | Panuveitis of right eye |
|  | 36676857 | Retinal dystrophy with inner retinal dysfunction and ganglion cell anomalies |
|  | 36676800 | Idiopathic posterior uveitis |
|  | 36676639 | Aphonia, deafness, retinal dystrophy, bifid halluces, intellectual disability syndrome |
|  | 36676632 | Progressive retinal dystrophy due to retinol transport defect |
|  | 36676439 | Infantile onset panniculitis with uveitis and systemic granulomatosis |
|  | 36675148 | Autosomal recessive leukoencephalopathy, ischemic stroke, retinitis pigmentosa syndrome |
|  | 36675066 | Microcornea, rod-cone dystrophy, cataract, posterior staphyloma syndrome |
|  | 36674817 | Occult macular dystrophy |
|  | 36674762 | Retinal macular dystrophy type 2 |
|  | 36674715 | Familial benign flecked retina |
|  | 36674513 | Microphthalmia, retinitis pigmentosa, foveoschisis, optic disc drusen syndrome |
|  | 35625724 | Bilateral iritis due to diabetes mellitus |
|  | 35625723 | Iritis of left eye due to diabetes mellitus |
|  | 35625722 | Iritis of right eye due to diabetes mellitus |
|  | 35624273 | Idiopathic panuveitis |
|  | 35622946 | Kandori fleck retina syndrome |
|  | 35622786 | Biallelic RPE65 mutation associated retinal dystrophy |
|  | 35622759 | Fundus albipunctatus |
|  | 35622071 | Best vitelliform macular dystrophy |
|  | 4339018 | Usher syndrome type 1 |
|  | 4339017 | X-linked retinitis pigmentosa heterozygote |
|  | 4339016 | X-linked retinitis pigmentosa |
|  | 4339015 | Autosomal recessive retinitis pigmentosa |
|  | 4339014 | Adult vitelliform macular dystrophy |
|  | 4338898 | Macular retinoschisis |
|  | 4336006 | Progressive rod dystrophy |
|  | 4335988 | Bacterial chorioretinitis |
|  | 4335981 | Anterior uveitis idiopathic |
|  | 4335593 | Pattern dystrophy of macula |
|  | 4334873 | Toxocara chorioretinitis |
|  | 4334251 | Usher syndrome type 2 |
|  | 4334250 | Autosomal dominant retinitis pigmentosa |
|  | 4334249 | Bull's eye macular dystrophy |
|  | 4334242 | Macular and peripheral retinoschisis |
|  | 4334134 | Onchocerca chorioretinitis |
|  | 4334133 | Birdshot chorioretinitis |
|  | 4327334 | Infection by Ophryoscolex |
|  | 4323630 | Uveitis due to ranibizumab |
|  | 4322865 | Congenital syphilitic choroiditis |
|  | 4319585 | Phacotoxic uveitis |
|  | 4319476 | Granulomatous chorioretinitis |
|  | 4319475 | Sclerouveitis |
|  | 4318987 | Phacoantigenic uveitis |
|  | 4318681 | Granulomatous choroiditis |
|  | 4313156 | Juvenile retinoschisis |
|  | 4311391 | Uveitis-rheumatoid arthritis syndrome |
|  | 4303512 | Tubulointerstitial nephritis with uveitis syndrome |
|  | 4294834 | Choroidal retinal neovascularization |
|  | 4291313 | Subacute cyclitis |
|  | 4289290 | Stargardt's disease |
|  | 4274964 | Iritis |
|  | 4273853 | Subacute anterior uveitis |
|  | 4265441 | Herpes simplex keratouveitis |
|  | 4259504 | Iritis with ulcerative colitis |
|  | 4256466 | Iritis in Behcet's syndrome |
|  | 4256465 | Uveitis due to leptospirosis |
|  | 4253623 | Intraocular lens associated postoperative inflammation |
|  | 4253620 | Iritis with Crohn's disease |
|  | 4253619 | Panuveitis in Behcet's syndrome |
|  | 4252044 | Lyme uveitis |
|  | 4240613 | Retinitis pigmentosa-deafness syndrome |
|  | 4234762 | Parasitic chorioretinitis |
|  | 4231849 | Vitelliform dystrophy |
|  | 4230371 | Fungal chorioretinitis |
|  | 4230369 | Fungal choroiditis |
|  | 4230365 | Parasitic choroiditis |
|  | 4227968 | Cryptococcus neoformans choroiditis |
|  | 4221343 | Herpetic iridocyclitis |
|  | 4220924 | Cryptococcal choroiditis |
|  | 4220916 | Human T-cell Lymphoma Virus Type -1 associated uveitis |
|  | 4220147 | Ocular histoplasmosis syndrome |
|  | 4218993 | Mycobacterium avium intracellulare group choroiditis |
|  | 4218925 | Retinitis pigmentosa-deafness-ataxia syndrome |
|  | 4218326 | Traumatic iritis |
|  | 4217839 | Subretinal fibrosis and uveitis syndrome |
|  | 4215673 | Cryptococcal chorioretinitis |
|  | 4215212 | Toxocara endophthalmitis |
|  | 4214659 | Multifocal choroiditis |
|  | 4214636 | Metipranolol-induced anterior uveitis |
|  | 4213657 | Propionibacterium acnes endophthalmitis |
|  | 4213641 | Chorioretinitis with coccidioidmycosis |
|  | 4212457 | Pamidronic acid-induced uveitis |
|  | 4212319 | Multifocal choroiditis and panuveitis syndrome |
|  | 4211939 | Anterior uveitis in juvenile idiopathic arthritis |
|  | 4211649 | Cidofovir-induced anterior uveitis |
|  | 4210871 | Inactive central serous retinopathy with focal retinal pigment epithelial detachment |
|  | 4210137 | Variant central serous chorioretinopathy |
|  | 4210136 | Multiple evanescent white dot syndrome |
|  | 4210135 | Punctate inner choroidopathy |
|  | 4210130 | Multifocal inner choroiditis |
|  | 4208204 | Idiopathic choroiditis |
|  | 4208203 | Bietti's crystalline retinopathy |
|  | 4208202 | North Carolina macular dystrophy |
|  | 4208201 | Chronic central serous chorioretinopathy |
|  | 4200131 | Peripapillary choroidal neovascular membrane |
|  | 4200130 | Extramacular choroidal neovascular membrane |
|  | 4200129 | Occult choroidal neovascular membrane |
|  | 4200128 | Classic choroidal neovascular membrane |
|  | 4199038 | Chronic central serous retinopathy with diffuse retinal pigment epithelial detachment |
|  | 4198347 | Progressive cone-rod dystrophy |
|  | 4198124 | Inactive toxoplasmosis chorioretinitis |
|  | 4198123 | Acute toxoplasmosis chorioretinitis |
|  | 4198122 | Post-traumatic uveitis |
|  | 4198121 | Tuberculous uveitis |
|  | 4197155 | Intermediate uveitis |
|  | 4197154 | Posterior cyclitis |
|  | 4196128 | Rod dystrophy |
|  | 4196116 | Tertiary syphilitic chorioretinitis |
|  | 4196110 | Acute central serous chorioretinopathy |
|  | 4195496 | Central serous retinopathy with small retinal pigment epithelial detachment |
|  | 4195495 | Acute central serous retinopathy with subretinal fluid |
|  | 4195054 | Syphilitic chorioretinitis |
|  | 4195053 | Sarcoid chorioretinitis |
|  | 4195051 | Inactive central serous chorioretinopathy |
|  | 4195048 | Cone dystrophy |
|  | 4194320 | Idiopathic choroidal neovascular membrane |
|  | 4194295 | Serpiginous choroiditis |
|  | 4194237 | Uveitis-glaucoma-hyphema syndrome |
|  | 4190954 | Sarcoid uveitis |
|  | 4190952 | Rifabutin-induced anterior uveitis |
|  | 4189317 | Lepromatous anterior uveitis |
|  | 4188694 | White dot syndrome |
|  | 4186741 | Brucellosis uveitis |
|  | 4185737 | Heerfordt's syndrome |
|  | 4183040 | Posterior uveitis |
|  | 4182267 | Gouty iritis |
|  | 4176864 | Acute anterior uveitis |
|  | 4174680 | Tuberculous disseminated chorioretinitis |
|  | 4173308 | Acute cyclitis |
|  | 4173026 | Hereditary macular dystrophy |
|  | 4171180 | Fundus flavimaculatus |
|  | 4166416 | Retinochoroidopathy |
|  | 4166240 | Chronic iridocyclitis in young girls |
|  | 4156419 | Keratouveitis |
|  | 4154710 | Generalized disseminated choroiditis AND chorioretinitis with acute necrosis |
|  | 4152064 | Postoperative uveitis |
|  | 4146105 | Non-infectious anterior uveitis |
|  | 4143364 | Progressive cone dystrophy |
|  | 4132792 | Anterior uveitis |
|  | 4132503 | Drug-induced uveitis |
|  | 4132502 | Lens-induced uveitis |
|  | 4132495 | Iritis in psoriatic arthritis |
|  | 4132493 | Infective uveitis |
|  | 4109415 | Secondary infected iridocyclitis |
|  | 4109410 | Disseminated posterior pole chorioretinitis |
|  | 4109407 | Peripheral focal retinochoroiditis |
|  | 4109112 | Oguchi's disease |
|  | 4108968 | Vogt-Koyanagi-Harada disease |
|  | 4108959 | Tapetoretinal dystrophy |
|  | 4108958 | Leber's amaurosis |
|  | 4108957 | Dominant drusen |
|  | 4108956 | Sorsby's fundus dystrophy |
|  | 4104213 | Disseminated peripheral chorioretinitis |
|  | 4104211 | Focal juxtapapillary choroiditis |
|  | 4104039 | Acute iritis |
|  | 4103381 | Retinitis pigmentosa |
|  | 4102657 | Hyaline retinal dystrophy |
|  | 4102654 | Hereditary retinal dystrophies in lipidoses |
|  | 4102176 | Iritis due to diabetes mellitus |
|  | 4102049 | General disseminated chorioretinitis |
|  | 4102047 | Juxtapapillary focal chorioretinitis |
|  | 4101333 | HSMN IV |
|  | 4099594 | Disseminated choroiditis |
|  | 4091179 | Tuberculous chronic iridocyclitis |
|  | 4090114 | Toxoplasmosis chorioretinitis |
|  | 4087293 | Tuberculous chorioretinitis |
|  | 4072340 | Herpes simplex iritis |
|  | 4060820 | Focal choroiditis |
|  | 4042904 | Choroiditis |
|  | 4032140 | Subacute iritis |
|  | 4028363 | Uveitis |
|  | 765216 | Chronic inflammation of uveal tract of bilateral eyes |
|  | 765078 | Retinitis pigmentosa of right eye |
|  | 765069 | Iridocyclitis of right eye |
|  | 765063 | Iritis of bilateral eyes |
|  | 762971 | Secondary infective uveitis |
|  | 761384 | Retinitis pigmentosa of bilateral eyes |
|  | 761383 | Retinitis pigmentosa of left eye |
|  | 761327 | Uveitis-hyphema-glaucoma syndrome of left eye |
|  | 761326 | Uveitis-hyphema-glaucoma syndrome of right eye |
|  | 761325 | Iridocyclitis of bilateral eyes |
|  | 761324 | Iridocyclitis of left eye |
|  | 761323 | Iritis of right eye caused by herpes simplex virus |
|  | 761322 | Iritis of left eye caused by herpes simplex virus |
|  | 761321 | Iridocyclitis of left eye due to herpes zoster |
|  | 761320 | Iridocyclitis of right eye due to herpes zoster |
|  | 761319 | Iridocyclitis of left eye caused by human herpes simplex virus |
|  | 761318 | Iridocyclitis of right eye caused by human herpes simplex virus |
|  | 761317 | Chronic inflammation of uveal tract of right eye |
|  | 761316 | Chronic inflammation of uveal tract of left eye |
|  | 761315 | Chronic endophthalmitis of right eye |
|  | 761314 | Chronic endophthalmitis of left eye |
|  | 761313 | Chorioretinitis of right eye caused by Toxoplasma gondii |
|  | 761312 | Chorioretinitis of bilateral eyes caused by Toxoplasma gondii |
|  | 761311 | Chorioretinitis of left eye caused by Toxoplasma gondii |
|  | 761192 | Iritis of left eye |
|  | 761191 | Iritis of right eye |
|  | 761144 | Bilateral age-related exudative degeneration of macula |
|  | 761143 | Age-related exudative macular degeneration of left eye |
|  | 761142 | Age-related exudative macular degeneration of right eye |
|  | 444446 | Gonococcal iridocyclitis |
|  | 443895 | Retinal dystrophy in systemic lipidosis |
|  | 441283 | Recurrent iridocyclitis |
|  | 440716 | Infectious secondary iridocyclitis |
|  | 440715 | Parasitic endophthalmitis |
|  | 440634 | Herpes simplex iridocyclitis |
|  | 439736 | Focal chorioretinitis due to acquired toxoplasmosis |
|  | 439731 | Secondary syphilitic iridocyclitis |
|  | 439689 | Retinal dystrophy in cerebroretinal lipidosis |
|  | 439671 | Uveitis due to secondary syphilis |
|  | 439300 | Secondary non-infected iridocyclitis |
|  | 439299 | Chronic iridocyclitis due to another disorder |
|  | 439017 | Peripheral focal choroiditis AND chorioretinitis |
|  | 439014 | Panuveitis |
|  | 438971 | Syphilitic disseminated retinochoroiditis |
|  | 438961 | Herpes zoster iridocyclitis |
|  | 438751 | Lens-induced iridocyclitis |
|  | 438744 | Acute posterior multifocal placoid pigment epitheliopathy |
|  | 438740 | Peripheral focal retinitis AND retinochoroiditis |
|  | 438739 | Sympathetic uveitis |
|  | 438422 | Primary iridocyclitis |
|  | 437850 | Focal chorioretinitis |
|  | 436967 | Juxtapapillary focal choroiditis AND chorioretinitis |
|  | 436393 | Focal choroiditis AND chorioretinitis of other posterior pole |
|  | 435541 | Generalized disseminated choroiditis AND chorioretinitis |
|  | 434932 | Fuchs' heterochromic cyclitis |
|  | 434927 | Disseminated choroiditis AND chorioretinitis, posterior pole |
|  | 434926 | Iridocyclitis |
|  | 434638 | Paramacular focal retinitis AND retinochoroiditis |
|  | 434348 | Acute and subacute iridocyclitis |
|  | 434274 | Secondary syphilitic chorioretinitis |
|  | 434033 | Chorioretinitis |
|  | 434029 | Chronic anterior uveitis |
|  | 434026 | Pars planitis |
|  | 433762 | Disseminated chorioretinitis |
|  | 433757 | Peripheral disseminated choroiditis AND chorioretinitis |
|  | 433187 | Cyclitis |
|  | 432908 | Glaucomatocyclitic crisis |
|  | 432632 | Noninfectious secondary iridocyclitis |
|  | 432631 | Metastatic disseminated retinitis AND retinochoroiditis |
|  | 432630 | Juxtapapillary focal retinitis AND retinochoroiditis |
|  | 380711 | Hyaline dystrophy of Bruch's membrane |
|  | 377270 | Hereditary retinal dystrophy |
|  | 376966 | Exudative age-related macular degeneration |
|  | 374936 | Achromatopsia |
|  | 374353 | Congenital stationary night blindness |
|  | 373772 | Vitreoretinal dystrophy |
|  | 372894 | Central serous chorioretinopathy |

**Supplementary Table S3. Baseline characteristic of patients treated with intravitreal anti-vascular endothelial growth factors or steroids for macular edema following retinal vein occlusion**

| **Characteristic** | **Count** | **% (n = 3,286)** |
| --- | --- | --- |
| **Age group** |  |  |
| 35 - 39 | 83 | 2.53 |
| 40 - 44 | 140 | 4.26 |
| 45 - 49 | 244 | 7.43 |
| 50 - 54 | 355 | 10.8 |
| 55 - 59 | 473 | 14.39 |
| 60 - 64 | 439 | 13.36 |
| 65 - 69 | 434 | 13.21 |
| 70 - 74 | 410 | 12.48 |
| 75 - 79 | 351 | 10.68 |
| 80 - 84 | 201 | 6.12 |
| 85 - 89 | 74 | 2.25 |
| **Gender: female** | 1768 | 53.8 |
| **Race** |  |  |
| race = Unknown | 58 | 1.77 |
| race = Korean | 3224 | 98.11 |
| **Medical history: General** |  |  |
| Diabetes mellitus | 223 | 6.79 |
| Gastroesophageal reflux disease | 43 | 1.31 |
| Hyperlipidemia | 78 | 2.37 |
| Hypertensive disorder | 375 | 11.41 |
| Osteoarthritis | 33 | 1 |
| Renal impairment | 53 | 1.61 |
| Visual system disorder | 2241 | 68.2 |
| **Medical history: Cardiovascular disease** |  |  |
| Cerebrovascular disease | 74 | 2.25 |
| Coronary arteriosclerosis | 39 | 1.19 |
| Heart disease | 147 | 4.47 |
| Ischemic heart disease | 41 | 1.25 |
| Venous thrombosis | 1399 | 42.57 |
| **Medical history: Neoplasms** |  |  |
| Malignant neoplastic disease | 81 | 2.47 |

**
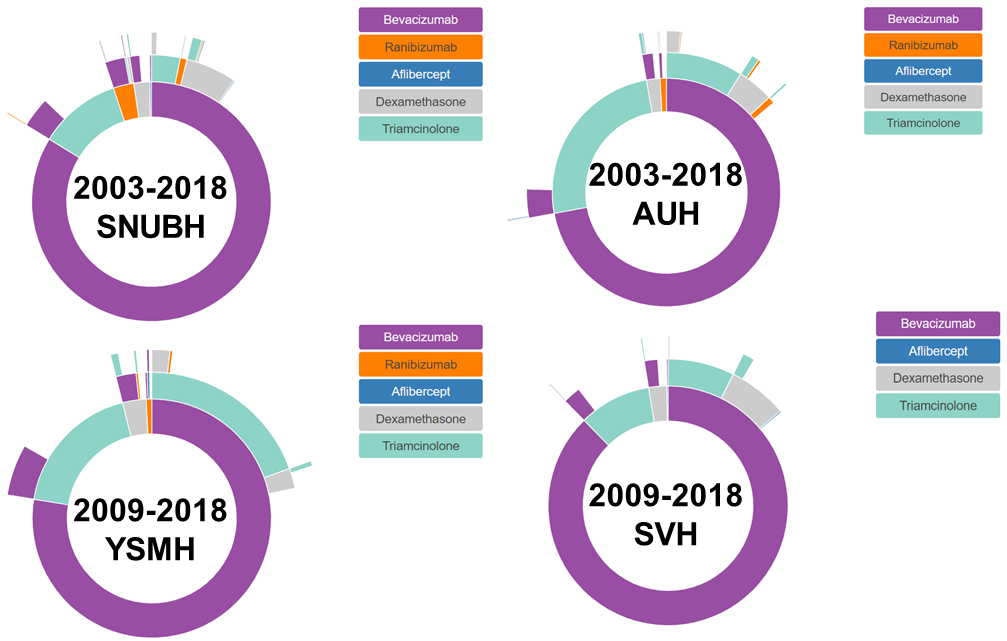
**

**Supplementary Figure S1. Sunburst diagrams of treatment pathways for macular edema following retinal vein occlusion at each center**

Abbreviations: SNUBH, Seoul National University Bundang Hospital; AUH, Ajou University Hospital; YSMH, Yeoeuido Saint Mary’s Hospital; SVH, Saint Vincent’s Hospital

**
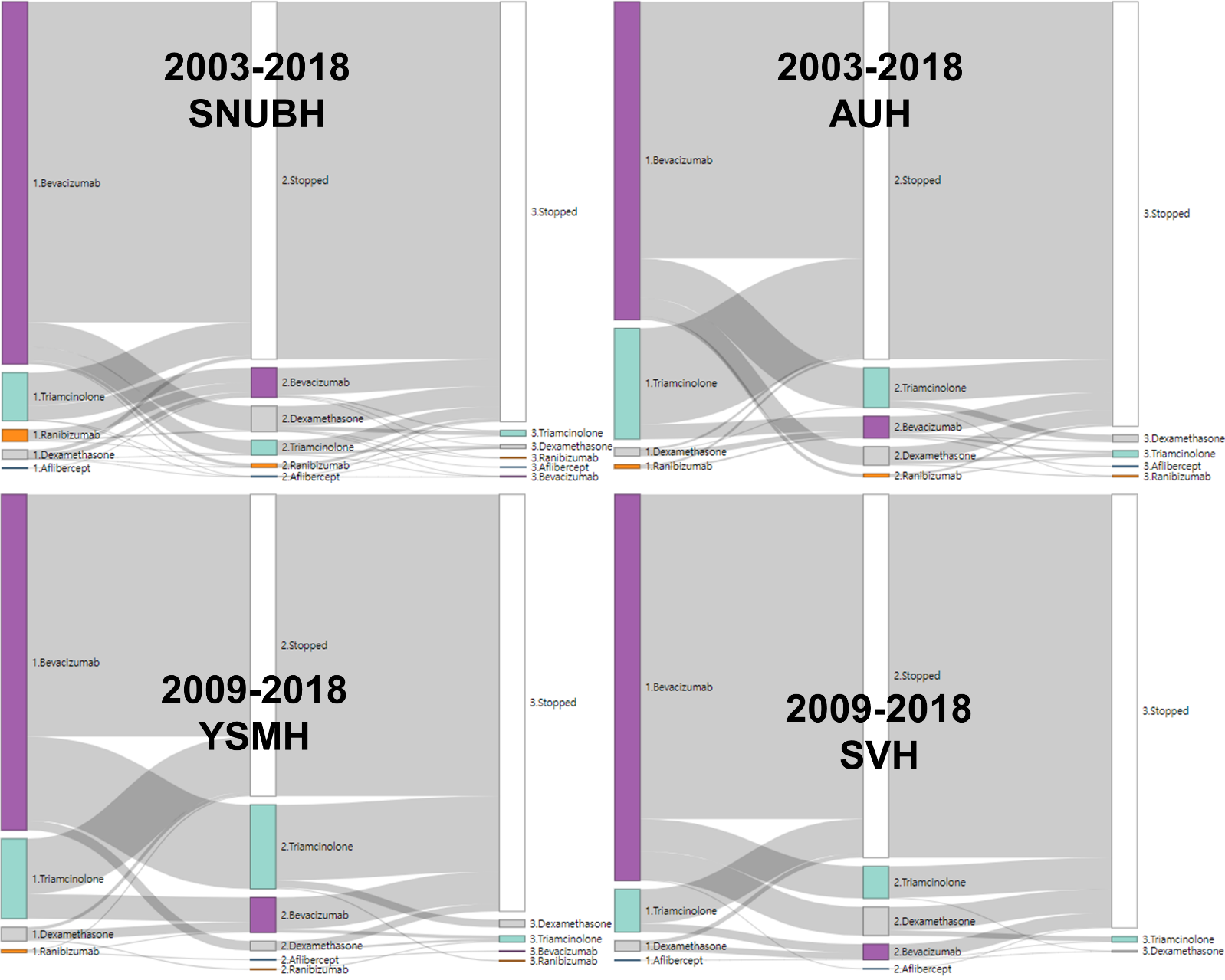
**

**Supplementary Figure S2. Sankey diagrams of treatment pathways for macular edema following retinal vein occlusion at each center**

Abbreviations: SNUBH, Seoul National University Bundang Hospital; AUH, Ajou University Hospital; YSMH, Yeoeuido Saint Mary’s Hospital; SVH, Saint Vincent’s Hospital
